# Sex differences in post-stroke cognitive decline: A population-based longitudinal study of nationally representative data

**DOI:** 10.1371/journal.pone.0268249

**Published:** 2022-05-06

**Authors:** Abdulaziz T. Bako, Thomas Potter, Jonika Tannous, Alan P. Pan, Carnayla Johnson, Eman Baig, Brian Downer, Farhaan S. Vahidy

**Affiliations:** 1 Center for Outcomes Research, Houston Methodist, Houston, TX, United States of America; 2 Department of Nutrition, Metabolism & Rehabilitation Sciences, University of Texas Medical Branch, Galveston, TX, United States of America; 3 Houston Methodist Neurological Institute, Houston, TX, United States of America; 4 Department of Population Health Sciences, Weill Cornell Medicine, New York, NY, United States of America; Auburn University, UNITED STATES

## Abstract

**Background:**

Sex differences in post-stroke cognitive decline have not been systematically evaluated in a nationally representative cohort. We use a quasi-experimental design to investigate sex differences in rate of post-stroke cognitive decline.

**Methods:**

Utilizing the event study design, we use the Health and Retirement Study (HRS) data (1996–2016) to evaluate the differences (percentage points [95% Confidence interval]) in the rate of change in cognitive function, measured using the modified version of the Telephone Interview for Cognitive Status (TICS-m) score, before and after incident stroke, and among patients with and without incident stroke. We estimated this event study model for the overall study population and separately fit the same model for male and female participants.

**Results:**

Of 25,872 HRS participants included in our study, 14,459 (55.9%) were females with an overall mean age (SD) of 61.2 (9.3) years. Overall, 2,911 (11.3%) participants reported experiencing incident stroke. Participants with incident stroke (vs. no stroke) had lower baseline TICS-m score (15.6 vs. 16.1). Among participants with incident stroke, the mean pre-stroke TICS-m score was higher than the mean post-stroke TICS-m score (14.9 vs. 12.7). Event study revealed a significant short-term acceleration of cognitive decline for the overall population (4.2 [1.7–6.6] percentage points, p value = 0.001) and among female participants (5.0 [1.7–8.3] percentage points, p value = 0.003). We, however, found no evidence of long-term acceleration of cognitive decline after stroke. Moreover, among males, incident stroke was not associated with significant changes in rate of post-stroke cognitive decline.

**Conclusion:**

Females, in contrast to males, experience post-stroke cognitive deficits, particularly during early post-stroke period. Identifying the sex-specific stroke characteristics contributing to differences in post stroke cognitive decline may inform future strategies for reducing the burden of post-stroke cognitive impairment and dementia.

## Introduction

Females have been reported to have higher prevalence of dementia [1,2], faster rate of age-associated cognitive decline [1,2], higher lifetime risk of cerebrovascular disease and stroke, as well as worse post-stroke functional outcomes [3–5], compared to males. Despite these remarkable sex differences, trajectories of post-stroke cognitive decline have not been systematically characterized among females and males, particularly so in a nationally representative cohort.

Moreover, prior studies investigating the association between stroke and cognitive decline are limited by a lack of control population (with no prior history of stroke) and/or failure to account for pre-stroke cognitive trajectories [6–9]. Also, evidence on the long-term association between a clinically overt stroke syndrome and rate of post-stroke cognitive decline, controlling for pre-stroke cognitive function, has largely been inconclusive with some authors reporting significant short-term decline in cognitive function followed by accelerated rate of post-stroke cognitive decline, and other studies reporting only a short-term decline in cognitive function without long-term acceleration of cognitive decline [6,10–13]. Identification of modifiable risk factors contributing to sex differences in cognitive decline may inform future cognitive impairment and dementia prevention strategies and guide development of targeted therapies.

In this study, we used a quasi-experimental design to characterize the short and long-term post-stroke changes in the overall and sex-disaggregated rates of cognitive decline while accounting for pre-stroke cognitive function, in a nationally representative cohort.

## Materials and methods

### Ethics statements

Study subjects include participants in the health and retirement study (HRS) between 1996 and 2016. Participants were provided with written informed consent prior to each interview. The HRS data is de-identified and publicly available upon request and completion of required online registration[14]; therefore, institutional review board approval for this research is not required by the Houston Methodist Hospital. Further details on data availability and ethics statement are provided in S1 Appendix.

### Data source and study population

The HRS is a nationally representative biennial survey of community dwelling adults (aged ≥ 50 years) and their spouses in the United States (US) [14,15]. The HRS is sponsored by the National Institute on Aging (grant number NIA U01AG009740) and is conducted by the University of Michigan. As participants enter the survey at different time points, we considered participants’ baseline to be the first HRS survey wave they responded to. We excluded participants with less than 2 follow up observations, those aged less than 50 years, those with baseline history of stroke or a baseline cognitive impairment, defined as having a score of ≤ 6 on the modified version of the Telephone Interview for Cognitive Status (TICS-m) [16]. These patients were excluded to avoid ‘floor effect’ of TICS-m, which may cause interpretational challenges in assessment of further cognitive decline. We also excluded participants’ survey waves that were completed by proxy respondents from our analysis because TICS-m is not assessed in proxy interviews. Furthermore, we censored participants’ interviews the first time they reported being diagnosed with Alzheimer’s disease or dementia, thereby allowing them to contribute to the outcome measures prior to reporting these diagnoses.

#### Dependent variable

Our primary outcome is the rate of change in cognitive function. HRS data captures cognitive function using TICS-m, which is a global test of cognition, modeled after the Mini-Mental State Examination (MMSE). TICS-m assesses cognitive domains that are commonly affected by stroke: memory, learning, and global cognition, and is validated for assessing cognitive function in adult community dwellers [17–19]. Moreover, TICS-m questionnaire items have been consistently captured in all the waves of HRS included in this study. TICS-m comprises 1) assessment of memory via testing participants’ ability for immediate and delayed recall of 10 noun-free words (0–20 points); 2) assessment of participants’ working memory using the serial 7 subtraction test (0–5 points); and 3) assessment of participants’ mental processing speed using counting backward test (0–2 points). We calculated the rate (in percentage points) of change in cognitive function by taking the difference between the natural log of TICS-m score on a given HRS wave and the natural log of TICS-m score in the preceding HRS wave and multiplying the difference by 100. In addition to the fact that this measure provides an approximation of the rate of change (growth or decline) in cognitive function from one period to the next, the use of log transformation removes the effect of time trend associated with the outcome [20]. Hence, in our study, this transformation stabilizes our model with respect to age-related decline in cognitive function.

#### Independent variable and other covariates

Our main exposure variable is self-reported history of first onset of stroke (incident stroke), which was derived from the binary (Yes/No) HRS questions: “Has a doctor ever told you that you had a stroke?” & “Since we last talked to you in [last interview date], has a doctor told you that you have a stroke?” We did not consider recurrent strokes in our event study analyses. Other variables used in our analysis include time-invariant covariates, such as age at baseline, sex, race / ethnicity and baseline level of education, and time-variant covariates, including participants’ current marital status; participants’ measure of depressive symptoms, obtained using the center for epidemiological studies—depression (CES-D) score (higher scores indicating more depressive symptoms); and self-reported history of hypertension, diabetes, cancer, heart disease, and psychiatric conditions. These covariates have been shown to be associated with post stroke cognitive decline and have been typically controlled for in prior studies investigating association of stroke with cognitive decline [12,13,21–23].

### Statistical analysis

We report participants’ overall and sex-disaggregated baseline descriptive characteristics using means (standard deviation [SD]) and proportions. Also, we report overall and sex-specific unadjusted incidence rates of stroke per 1,000 person years with 95% confidence intervals (CI). We also provide information on the number of participants, as well as frequency of incident stroke, per wave (S1 Table). We used an event study design to examine the effect of incident stroke on rate of cognitive decline. A detailed explanation of the event study design is available in S2 Appendix. Briefly, the event study design is a quasi-experimental staggered adoption design, similar to the difference-in-differences (DID) design, that allows for comparison of pre- and post-exposure changes in outcome variable among exposed and unexposed. In the event study design, exposed subjects are required to have similar pre-treatment trends in the outcome variable as those who are unexposed, the so-called parallel trends assumption. If the assumption holds and important time-varying covariates are not omitted, the event study design provides causal estimates of the relationship between exposure and outcome variables. Additionally, event study design inherently controls for time-invariant confounders, such as baseline age, level of education, sex and race. Our event study model can be written as follows:

$$Cognitive\_Decline_{it}=\alpha +\beta 8{\left(Lag\;8\_plus\right)}_{it}+\beta 7{\left(Lag\;7\right)}_{it}+\beta 6{\left(Lag\;6\right)}_{it}+\beta 5{\left(Lag\;5\right)}_{it}+\beta 4{\left(Lag\;4\right)}_{it}+\beta 3{\left(Lag\;3\right)}_{it}+\beta 2{\left(Lag\;2\right)}_{it}+\gamma 0{\left(Lead\;0\right)}_{it}+\gamma 1{\left(Lead\;1\right)}_{it}+\gamma 2{\left(Lead\;2\right)}_{it}+\gamma 3{\left(Lead\;3\right)}_{it}+\gamma 4{\left(Lead\;4\right)}_{it}+\gamma 5{\left(Lead\;5\right)}_{it}+\gamma 6{\left(Lead\;6\right)}_{it}+\gamma 7{\left(Lead\;7\right)}_{it}+\gamma 8{\left(Lead\;8\_plus\right)}_{it}+X_{it}\Gamma +\mu_{i}+\lambda_{t}+\varepsilon_{it}$$

Where Cognitive_Decline_it_ is the rate of decline in cognitive function (in percentage points) for individual *i* at HRS wave *t*. Leads and lags were considered relative to the reference period, which is the HRS wave immediately preceding the wave in which stroke was self-reported. The reference period (Lag 1) is omitted in the model to capture the baseline difference between those with incident stroke and those without incident stroke. The coefficient of lag *t*, for example, captures the difference between the rate of cognitive decline in the reference period and the rate of cognitive decline t waves (periods) before incident stroke. Similarly, the coefficient of lead *t* represents the effect of incident stroke on the rate of cognitive decline t waves after the wave in which incident stroke was self-reported. Lead 0 represents the effect of incident stroke on rate of cognitive decline in the wave when incident stroke was reported. Of note, *Lag 8_plus* and *Lead 8_plus*, respectively, represent the accumulated effect of incident stroke on rate of cognitive decline 8 or more waves (16 or more years) before and after stroke. We considered the coefficient of Lead 0 to represent the short-term effect of stroke on TICS-m score and Lead 1 to Lead 8_Plus to represent the long-term effect of stroke on TICS-m score. Therefore, we formally tested the joint significance of the long-term coefficients simultaneously using the F test. A combination of non-significant F test and non-significant long-term coefficients (coefficients of Lead 1 to Lead 8_Plus) will indicate that incident stroke is not associated with sustained long-term acceleration of post-stroke cognitive decline.

Utilizing the *eventdd* package [24] in Stata (v.16) statistical software [25], we fit a series of event study models using the high definition fixed effects (HDFE) regression, with robust standard errors clustered at household level, to estimate changes in the rate of cognitive decline associated with incident stroke. Details of the *eventdd* package are discussed elsewhere [24]. We also provide a stylized example of how lead and lag variables are coded (*S3 Appendix*), as well as a Stata do-file detailing the implementation of our event study analysis (*S1 File*) We used 0.05 as the level of significance in all our analyses.

### Sensitivity analyses

We assessed for potential attrition bias by repeating our analysis while sequentially increasing the number of follow-ups required for participants to be included in the analyses from 2 to 3, 4, 5, and 6. Also, to minimize imbalance in our dataset, we restricted the analysis to only HRS participants who responded to the 1996 survey.

## Results

### Baseline characteristics

**Fig 1:**
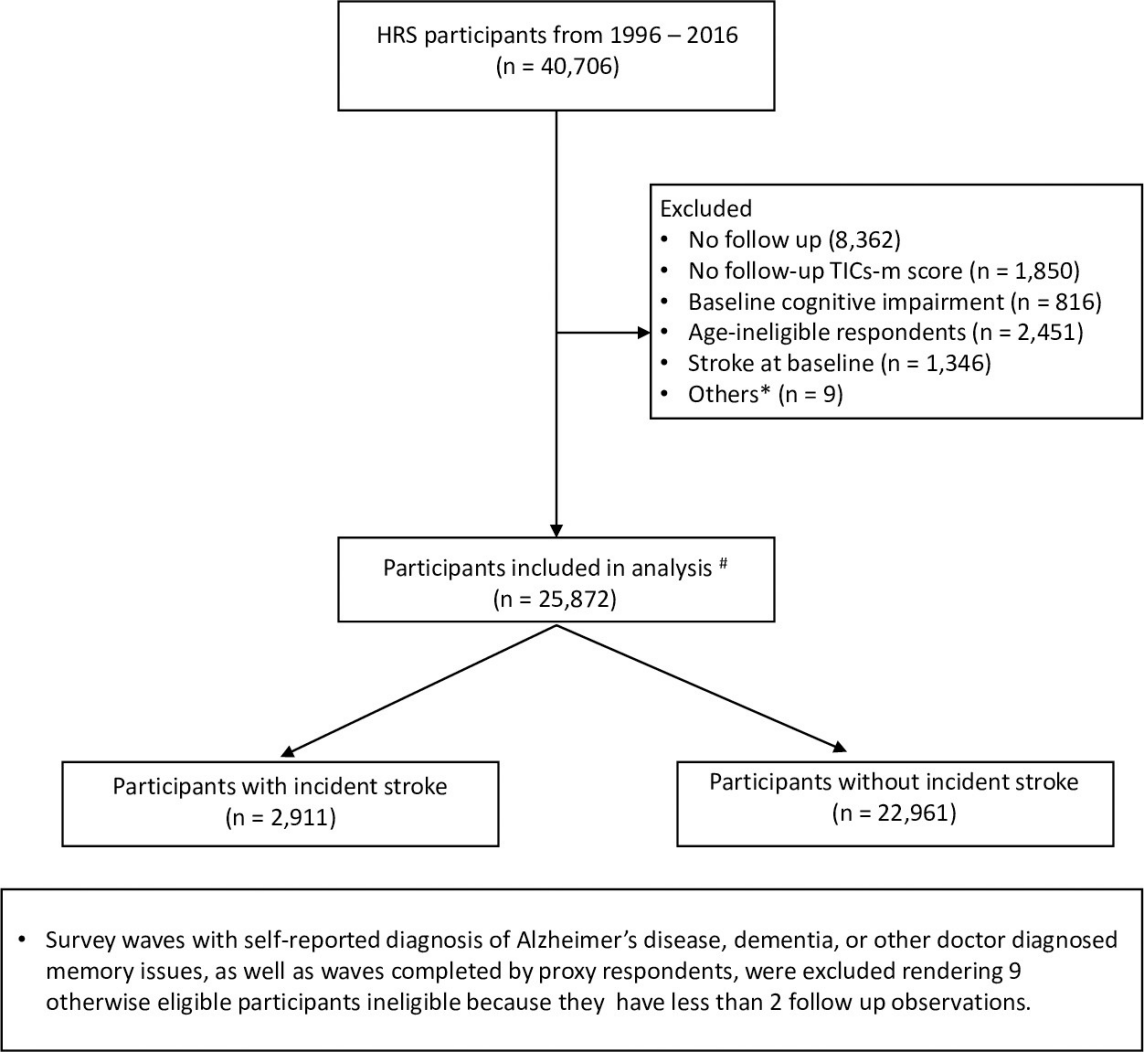
Participant selection flow chart.

The final analytic sample included 25,872 HRS participants (Fig 1) with an overall mean (SD) age of 61.2 (9.3) years. Among these participants 14,459 (55.9%) were females and 2,911 (11.3%) reported having incident stroke during follow-up (*Table 1*). Compared to those without incident stroke, participants with incident stroke were significantly older (60.7 vs. 65.3 years, p-value < 0.001); had a significantly higher burden of hypertension (42.1% vs. 53.8%, p-value < 0.001), diabetes (12.7% vs. 17.6%, p-value < 0.001), cancer (8.1% vs. 9.8%, p-value = 0.005), heart disease (14.8% vs. 25.2%, p-value < 0.001) and psychiatric illness (12.6% vs. 15.1%, p-value < 0.001); and had a higher (worse) CES-D score (1.3 vs. 1.5, p-value < 0.001), at baseline. However, participants with self-reported incident stroke had significantly lower baseline years of education (12.5 years vs. 12.1 years, p-value < 0.001) and TICS-m score (16.1 vs. 15.6, p-value < 0.001). Among those who reported having stroke, mean TICS-m score during their pre-stroke period(s) was significantly higher, compared to the mean score in post-stroke period (14.9 vs. 12.7, p-value < 0.001).

**Table 1:** Participants’ baseline characteristics by stroke status.

Participant characteristics, n (%)	Overall (n = 25,872)		No Incident Stroke (n = 22,961)		With Incident Stroke (n = 2,911)		p-value
**Age, mean (SD)**	61.2	(9.3)	60.7	(9.2)	65.3	(9.5)	< 0.001
**Female**	14,459	(55.9)	12,811	(55.8)	1,648	(56.6)	0.402
**Race / ethnicity**							
**Non-Hispanic White**	17,985	(69.6)	15,827	(69.0)	2,158	(74.2)	< 0.001
**Non-Hispanic Black**	4,333	(16.8)	3,847	(16.8)	486	(16.7)	0.917
**Non- Hispanic other race**	682	(2.6)	638	(2.8)	44	(1.5)	< 0.001
**Hispanic**	2,839	(11.0)	2,617	(11.4)	222	(7.6)	< 0.001
**Marital status**							
**Never Married**	1,159	(4.6)	1,062	(4.7)	97	(3.4)	0.002
**Married or Partnered**	17,905	(70.8)	15,978	(71.2)	1,927	(68.4)	0.002
**Divorced or Separated**	3,232	(12.8)	2,939	(13.1)	293	(10.4)	< 0.001
**Widowed**	2,975	(11.8)	2,474	(11.0)	501	(17.8)	< 0.001
**Years of education at baseline, mean (SD)**	12.5	(3.2)	12.5	(3.2)	12.1	(3.1)	< 0.001
**Hypertension**	10,995	(43.4)	9,476	(42.1)	1,519	(53.8)	< 0.001
**Diabetes**	3,352	(13.2)	2,856	(12.7)	496	(17.6)	< 0.001
**Cancer**	2,103	(8.3)	1,828	(8.1)	275	(9.8)	0.003
**Heart Disease**	4,045	(16.0)	3,334	(14.8)	711	(25.2)	< 0.001
**Psychiatric Illness**	3,264	(12.9)	2,839	(12.6)	425	(15.1)	< 0.001
**CES-D Score, mean (SD)**	1.4	(1.9)	1.3	(1.9)	1.5	(2.0)	< 0.001
**TICS-m Score, mean (SD)**	16.1	(4.0)	16.1	(4.0)	15.6	(4.2)	< 0.001

Missing data: Race/ethnicity (33); marital status (601); hypertension (564); diabetes (563); cancer (566); heart disease (560); psychiatric conditions (569).

We observed incident stroke among 1,263 (11.1%) males and 1,648 (11.4%) females (p value = 0.402) over a mean (SD) of 11.2 (5.8) years of follow-up (Table 2). While males (vs. females) have a higher burden of diabetes (14.1% vs. 12.6%, p-value < 0.001) and heart disease (18.0% vs. 14.4%, p-value < 0.001), females have a significantly higher burden of cancer (9.4% vs. 6.9%, p-value < 0.001) and psychiatric conditions (15.9% vs. 9.1%, p-value < 0.001), as well as a higher (worse) mean CES-D score (1.5 vs. 1.2, p-value < 0.001) (*Table 2*). Overall, the crude incidence rate (CI) of stroke per 1,000 person-years was 9.0 (8.6–9.3). Among males, the crude incidence rate (CI) was 9.2 (8.7–9.7) per 1,000 person-years, and among females, it was 8.8 (8.4–9.3) per 1,000 person-years.

**Table 2:** Participants’ baseline characteristics by sex.

Participant characteristics	Male (n = 11,413)		Female (n = 14,459)		p-value
**Age, mean (SD)**	61.0	(9.0)	61.5	(9.6)	< 0.001
**Incident stroke (%)**	1,263	(11.1)	1,648	(11.4)	0.402
**Non-Hispanic Race and Ethnicity (%)**					
**Non-Hispanic White**	8,062	(70.8)	9,923	(68.7)	< 0.001
**Non-Hispanic Black**	1,713	(15.0)	2,620	(18.1)	< 0.001
**Non-Hispanic Other race**	331	(2.9)	351	(2.4)	0.018
**Hispanic**	1,289	(11.3)	1,550	(10.7)	0.138
**Marital status (%)**					
**Never Married**	473	(4.3)	686	(4.9)	0.024
**Married or Partnered**	9,045	(81.3)	8,860	(62.6)	< 0.001
**Divorced or Separated**	1,106	(9.9)	2,126	(15.0)	< 0.001
**Widowed**	499	(4.5)	2,476	(17.5)	< 0.001
**Years of education at baseline, mean (SD)**	12.6	(3.4)	12.3	(3.1)	< 0.001
**Hypertension**	4,749	(42.6)	6,246	(44.1)	0.022
**Diabetes**	1,571	(14.1)	1,781	(12.6)	< 0.001
**Cancer**	766	(6.9)	1,337	(9.4)	< 0.001
**Heart Disease**	2,008	(18.0)	2,037	(14.4)	< 0.001
**Psychiatric Illness**	1,008	(9.1)	2,256	(15.9)	< 0.001
**CES-D Score, mean (SD)**	1.2	(1.8)	1.5	(2.0)	< 0.001
**TICS-m Score, mean (SD)**	15.8	(3.9)	16.3	(4.1)	< 0.001

Missing data: Race/ethnicity (33); marital status (601); hypertension (564); diabetes (563); cancer (566); heart disease (560); psychiatric conditions (569).

### Event study estimates

**Fig 2:**
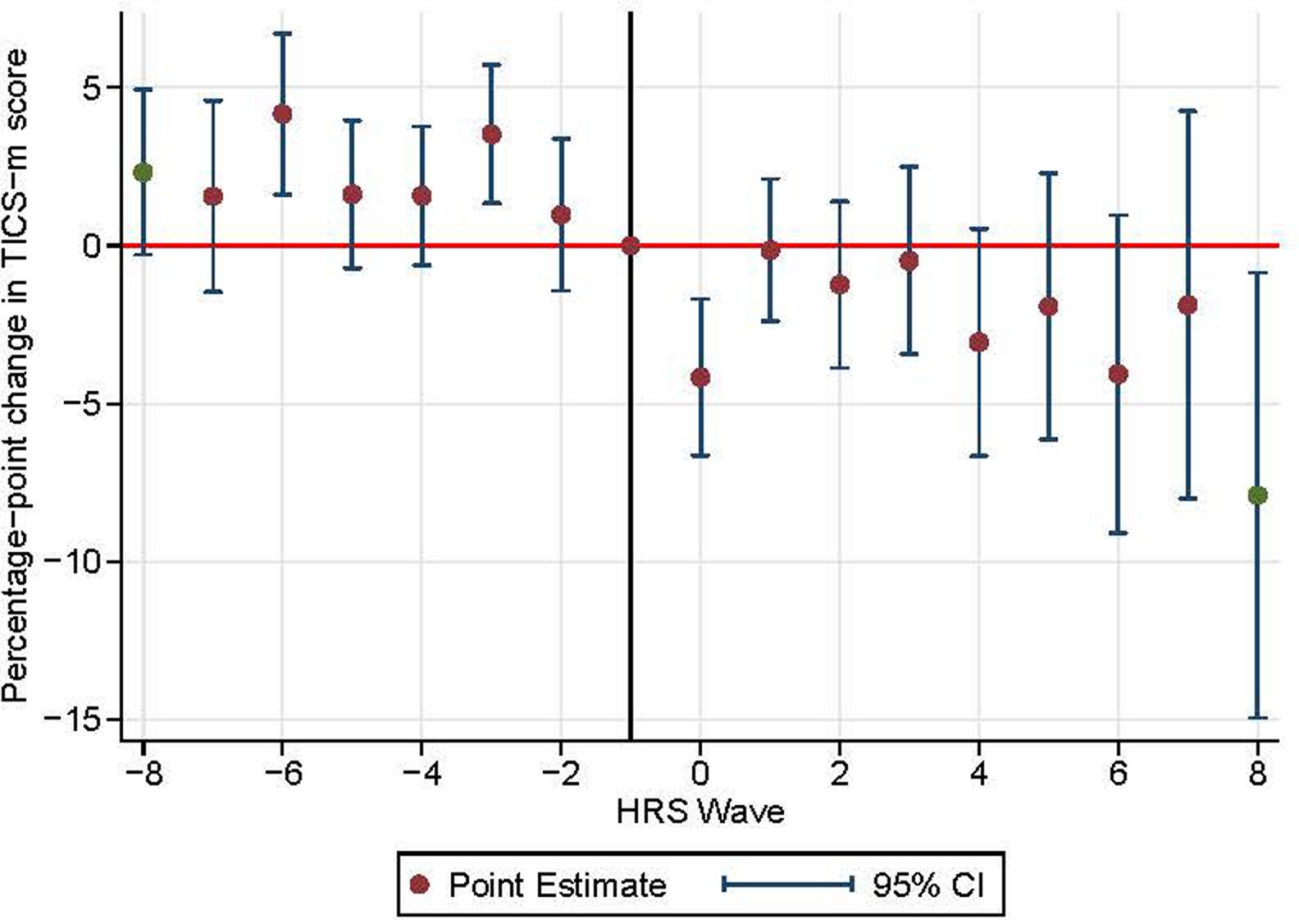
Event study plot for overall population.

In the overall population, we observed a significant short-term acceleration of cognitive decline in the HRS wave immediately following incident stroke. Specifically, cognitive function declined 4.2 (1.7–6.6, p value = 0.001) percentage points faster, relative to the reference period, in the overall population (S2 Table and Fig 2). However, rates of post stroke cognitive decline in the long-term periods after stroke incidence were not statistically different from the decline rate during the reference period (p-value for joint F test = 0.21).

**Fig 3:**
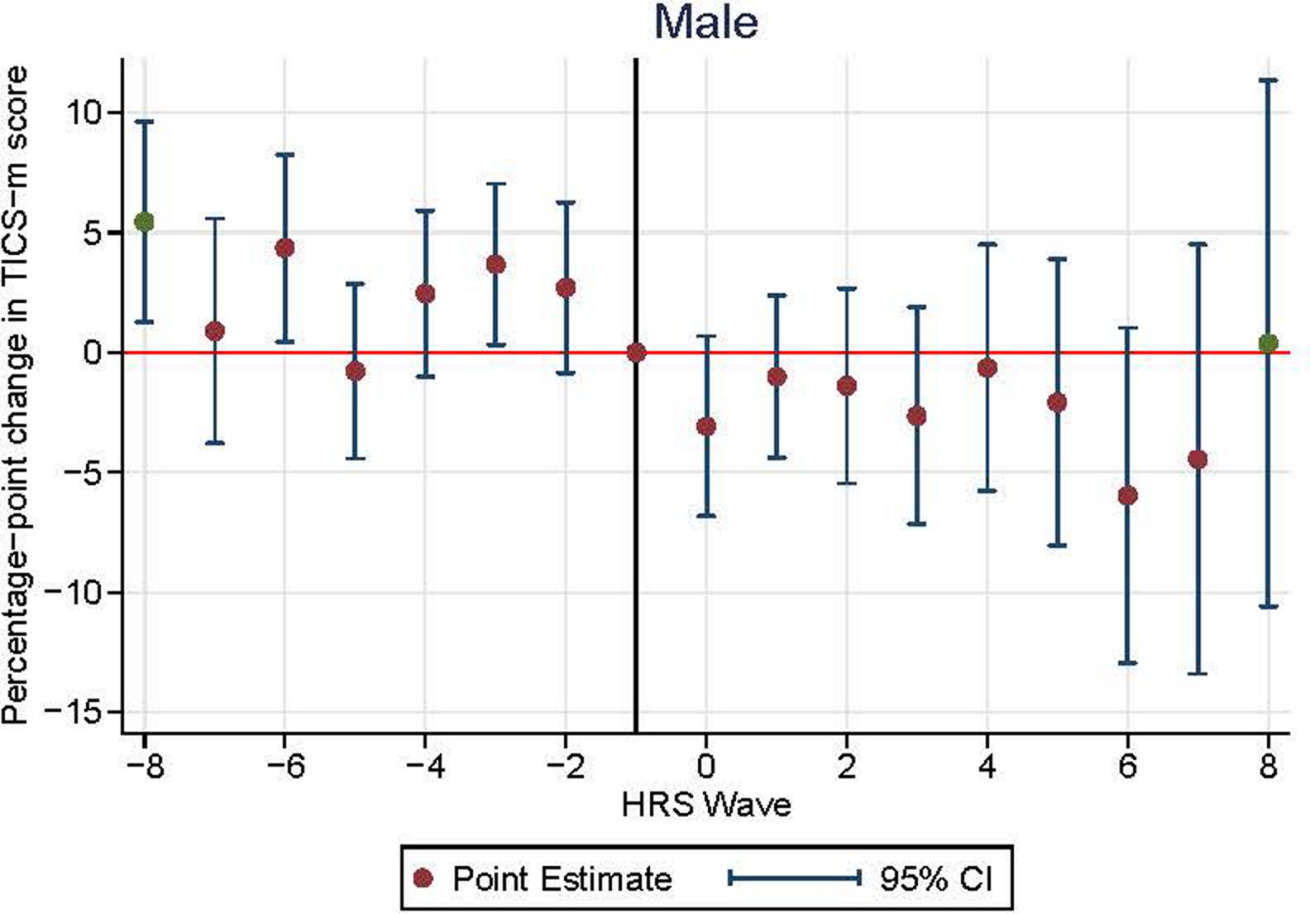
Event study plot for male participants.

**Fig 4:**
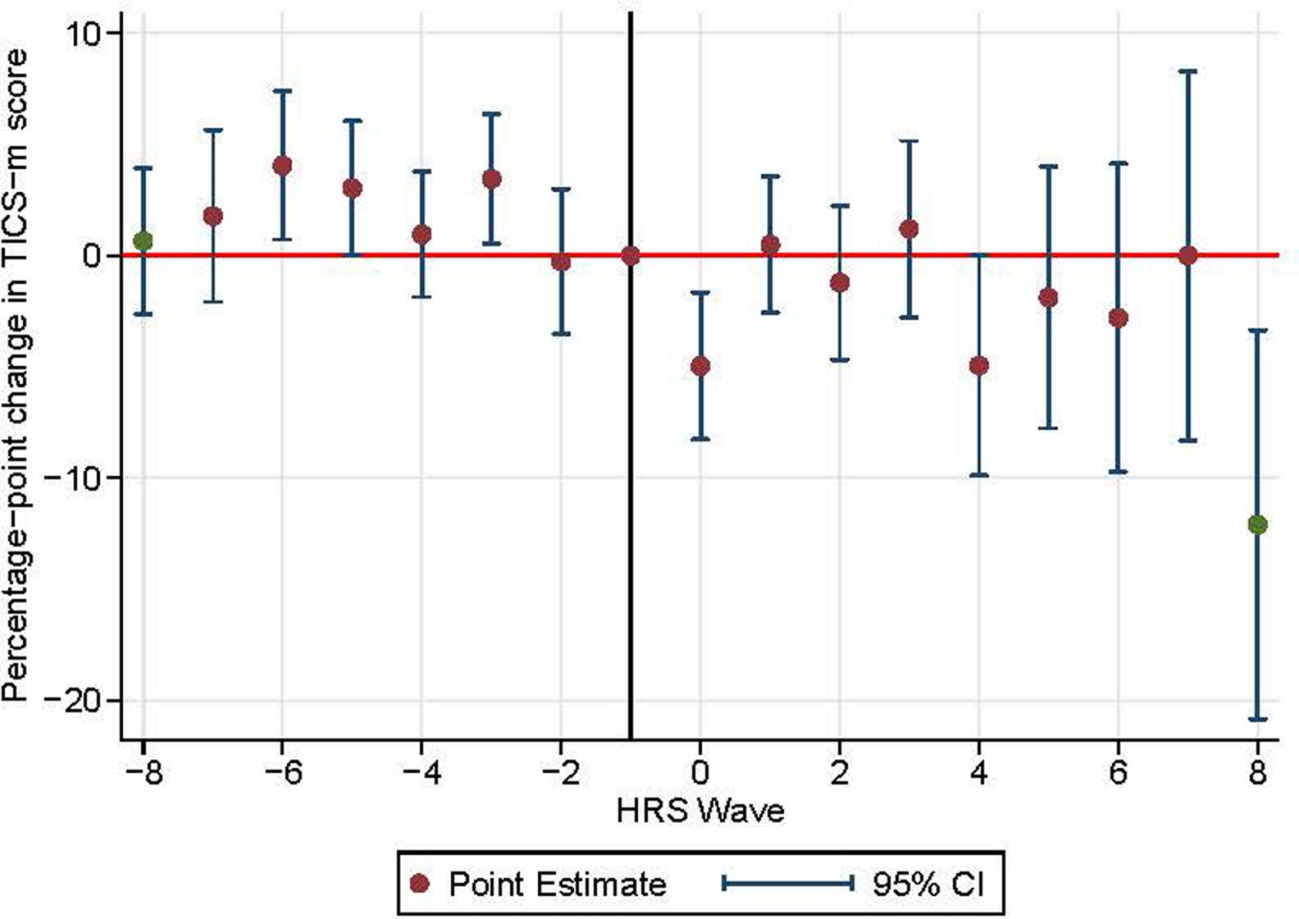
Event study plot for female participants.

Among male participants, the rates of cognitive decline in all post-stroke periods were not significantly different from the reference period rate (S2 Table and Fig 3). However, among females, cognitive function significantly declined 5.0 percentage points (1.7–8.3, p value = 0.003) faster in the period immediately following stroke incidence (S2 Table and Fig 4). Also, similar to the overall population, the rates of cognitive decline among females in the long-term post-stroke periods were not significantly different from the rate of decline in the reference period (p-value for joint F test = 0.11).

### Sensitivity analyses

Increasing the required number of follow-ups and limiting analysis to participants who responded to 1996 HRS survey did not significantly alter the magnitude and significance of event study coefficients for post-stroke periods (S1–S10 Figs).

### Missing data

We imputed participants’ baseline age using reported age in other survey waves, thereby obtaining complete information of patients’ age. However, participants with missing data (mainly comorbidities, marital status, or race/ethnicity data) constitute less than 3% of our total study population; consequently, we report our findings based on complete case analysis.

## Discussion

Using a nationally representative sample of community dwelling adults in the US, we found evidence of sex differences in the association of incident stroke with cognitive function. Our data suggests that incident stroke is associated with a short-term acceleration of cognitive decline in females but not in males. We also observed that incident stroke was not associated with long-term changes in cognitive function, as measured by TICS-m.

Prior studies, including Levine et al. [12,13], Wang et al. [10], and Lu et al. [11], have evaluated the association between incident stroke and cognitive decline; however, these studies did not systematically evaluate sex differences in post stroke cognitive decline. Although prior small studies have investigated sex differences in post stroke dementia and cognitive impairment [26–28], to our knowledge, this is the first study in the US that used a nationally representative cohort to evaluate sex differences in post stroke cognitive decline. After controlling for potential confounders that are known to be associated with both stroke and cognitive decline [29–31], our analyses indicate that incident stroke is associated with short-term acceleration of cognitive decline among females only. This finding provides evidence that sex modifies the association between stroke and cognitive function.

The pathophysiological mechanisms underlying sex differences in cognitive decline after acute brain insults, such as stroke, are not clearly understood. However, sex differences in the expression of brain-derived neurotrophic factor, which has been shown to affect memory, learning, and stroke severity through its actions on neuronal and synaptic plasticity, may potentially explain sex differences in post stroke cognitive decline [32–35]. Moreover, sex hormones, particularly progesterone, have been shown to increase the expression and signaling of brain-derived neurotrophic factor after ischemic stroke, with subsequent improvement in neurologic outcomes [36]. Further, it has been shown that differential expression of estradiol among different sexes may modulate sex differences in stroke severity [35,37,38]. However, further studies are needed to evaluate the biologic mechanisms driving sex differences in post stroke cognitive outcomes.

Also, the fact that males and females had similar frequency of incident stroke might suggest that sex differences in stroke severity, stroke type, stroke location or other stroke characteristics may explain the sex differences in post stroke cognitive decline that we observed in our study. Also, sex differences in post-stroke rehabilitation, and control of vascular risk factors, may explain the sex differences in post-stroke cognitive decline observed in this study. It is also possible that stroke causes a greater detrimental effect on cognitive function among females. However, our data are limited by lack of information regarding stroke subtype, location, or severity, as well as post-stroke rehabilitation and treatment details. Future studies should, therefore, further investigate how these factors may be contributing to sex differences in post-stroke cognitive decline.

Consistent with prior studies [6,12,13], our findings suggest that incident stroke is not associated with long term acceleration of cognitive decline after incident stroke. Of note, Levine et al. [13] observed, utilizing the REasons for Geographic And Racial Differences in Stroke (REGARDS) cohort, that stroke is associated with long term acceleration of decline in global cognition and executive function. However, the authors also found that stroke is not associated with long term acceleration of decline in short and long-term memory [13]. Similarly, Lu et al. and Wang et al. [10,11] reported significant short-term decrease in memory function after first and recurrent stroke but with no evidence of long-term acceleration of decline in memory. Also, Levine et al. [12], utilizing HRS data, found that incident stroke is associated with short term decline in cognitive function, measured using TICS-m score, but with no evidence of accelerated decline in cognitive function in the long-term periods after stroke. Given that our measure of cognition in this study, the TICS-m score, heavily relies on participants’ memory function; it may be logical to infer that incident stroke is not associated with accelerated deterioration of memory function in the long term, as documented in other studies [6,12].

Our study utilized a large nationally representative cohort of patients with stroke to investigate the association of incident stroke with cognitive decline, while adjusting for pre-stroke cognitive measures. This large sample size adds strengths to our findings. However, the fact that stroke is ascertained through self-report in our study, rather than using administrative or clinical data, may subject our findings to misclassification bias. Also, owing to its limited sensitivity in detecting decline in executive function [16,39], a cognitive domain that is known to be highly impacted by stroke [40], TICS-m may lack sensitivity to detecting decline in executive function after stroke among our study participants. Also, our study may be subject to attrition bias, which may result in underestimation cognitive decline if individuals with low cognitive function at baseline are more likely to die, require proxy interview, or drop out of the cohort. However, an analysis that accounted for attrition bias by sequentially increasing the number of follow-ups required for inclusion in the study from 2 to 3,4,5, and 6, consistent with Levine et al. [12,13], Wang et al. [10] and Salthouse [41], did not change our main findings.

## Conclusion

In a nationally representative cohort of community dwelling adults in the US, we found, after controlling for pre-stroke cognitive measures, that females, in contrast to males, experience post-stroke cognitive deficits, particularly during early post-stroke period. Identifying the sex-specific stroke characteristics contributing to differences in post-stroke cognitive decline may inform development of practice guidelines and targeted therapeutics for reducing sex disparities in post stroke cognitive impairment and dementia.

## Supporting information

S1 Fig Sensitivity analyses. Event study plot after excluding patients with less than 3 follow-ups (overall).
(EPS)

S2 Fig Sensitivity analyses. Event study plot after excluding patients with less than 3 follow-ups (disaggregated by sex).
(EPS)

S3 Fig Sensitivity analyses. Event study plot after excluding patients with less than 4 follow-ups (overall).
(EPS)

S4 Fig Sensitivity analyses. Event study plot after excluding patients with less than 4 follow-ups (disaggregated by sex).
(EPS)

S5 Fig Sensitivity analyses. Event study plot after excluding patients with less than 5 follow-ups (overall).
(EPS)

S6 Fig Sensitivity analyses. Event study plot after excluding patients with less than 5 follow-ups (disaggregated by sex).
(EPS)

S7 Fig Sensitivity analyses. Event study plot after excluding patients with less than 6 follow-ups (overall).
(EPS)

S8 Fig Sensitivity analyses. Event study plot after excluding patients with less than 6 follow-ups (disaggregated by sex).
(EPS)

S9 Fig Sensitivity analyses. Event study plot after limiting analysis to participants that responded to 1996 interview (overall).
(EPS)

S10 Fig Sensitivity analyses. Event study plot after limiting analysis to participants that responded to 1996 interview (disaggregated by sex).
(EPS)

S1 Table Number of participants and frequency of incident stroke per survey wave. (DOCX)

S2 Table Estimates of event study coefficient, overall and stratified by gender. (DOCX)

S3 Table Event study coefficients for other time-varying covariates. (DOCX)

S1 Appendix Details on data availability and ethics statement. (DOCX)

S2 Appendix Extended methods. (DOCX)

S3 Appendix A stylized example of variable coding for event study lags and leads. (DOCX)

S1 File Stata do-file. (DO)
