# Supplementary material for: Sex differences in post-stroke cognitive decline: A population-based longitudinal study of nationally representative data
Source: PLoS One. 2022 May 6;17(5):e0268249. doi: 10.1371/journal.pone.0268249 (PMC9075630; doi:10.1371/journal.pone.0268249)
Supplement: S1 Table — (DOCX) [file pone.0268249.s011.docx]

| **Survey wave** | **Number of participants** | **Number of participants with stroke (%)** |
| --- | --- | --- |
| Wave 1 (1996) | 13,175 | 0 (0) * |
| Wave 2 (1998) | 17,041 | 250 (1.5) |
| Wave 3 (2000) | 16,128 | 286 (1.8) |
| Wave 4 (2002) | 15,227 | 337 (2.3) |
| Wave 5 (2004) | 16,699 | 281 (1.7) |
| Wave 6 (2006) | 15,607 | 304 (2.0) |
| Wave 7 (2008) | 14,612 | 288 (2.0) |
| Wave 8 (2010) | 18,184 | 331 (1.9) |
| Wave 9 (2012) | 17,162 | 316 (1.9) |
| Wave 10 (2014) | 15,618 | 262 (1.7) |
| Wave 11 (2016) | 13,627 | 256 (1.9) |

**S3 Table: Number of participants and frequency of incident stroke per survey wave**

* Participants with stroke at or before baseline were excluded from analysis.
