# Supplementary material for: Sex differences in post-stroke cognitive decline: A population-based longitudinal study of nationally representative data
Source: PLoS One. 2022 May 6;17(5):e0268249. doi: 10.1371/journal.pone.0268249 (PMC9075630; doi:10.1371/journal.pone.0268249)
Supplement: S2 Table — (DOCX) [file pone.0268249.s012.docx]

S2 Table: Estimates of event study coefficient, overall and stratified by gender

| **Variable** | **Overall** | **Males** | **Females** |
| --- | --- | --- | --- |
| **Survey waves relative to time of incident stroke** |  |  |  |
| **8 or more waves before** | 2.32 (-0.29 – 4.92) | 5.45 (1.29 – 9.61) * | 0.65 (-2.64 – 3.94) |
| **7 waves before** | 1.56 (-1.46 – 4.58) | 0.90 (-3.79 – 5.58) | 1.78 (-2.08 – 5.65) |
| **6 waves before** | 4.17 (1.62 – 6.72) ** | 4.36 (0.46 – 8.26) * | 4.05 (0.72 – 7.38) * |
| **5 waves before** | 1.62 (-0.71 – 3.96) | -0.78 (-4.42 – 2.86) | 3.03 (0.01 – 6.04) * |
| **4 waves before** | 1.58 (-0.62 – 3.77) | 2.46 (-0.99 – 5.92) | 0.95 (-1.87 – 3.77) |
| **3 waves before** | 3.53 (1.33 – 5.72) ** | 3.68 (0.33 – 7.04) * | 3.45 (0.54 – 6.36) * |
| **2 waves before** | 0.97 (-1.43 – 3.37) | 2.71 (-0.84 – 6.27) | -0.27 (-3.52 – 2.99) |
| **1 wave before** | Reference | Reference | Reference |
| **0 wave after** | -4.16 (-6.64 – -1.69) ** | -3.08 (-6.83 – 0.68) | -4.97 (-8.27 – -1.66) ** |
| **1 wave after** | -0.15 (-2.40 – 2.11) | -1.01 (-4.40 – 2.38) | 0.49 (-2.58 – 3.56) |
| **2 waves after** | -1.24 (-3.87 – 1.39) | -1.39 (-5.46 – 2.67) | -1.22 (-4.68 – 2.23) |
| **3 waves after** | -0.48 (-3.45 – 2.49) | -2.64 (-7.15 – 1.87) | 1.19 (-2.77 – 5.15) |
| **4 waves after** | -3.06 (-6.66 – 0.53) | -0.64 (-5.77 – 4.50) | -4.94 (-9.87 – 0.00) |
| **5 waves after** | -1.93 (-6.14 – 2.28) | -2.08 (-8.04 – 3.88) | -1.88 (-7.77 – 4.00) |
| **6 waves after** | -4.07 (-9.10 – 0.96) | -5.96 (-12.95 – 1.03) | -2.80 (-9.72 – 4.13) |
| **7 waves after** | -1.88 (-8.00 – 4.24) | -4.45 (-13.40 – 4.49) | 0.00 (-8.30 – 8.30) |
| **8 or more waves after** | -7.90 (-14.94 – -0.86) * | 0.38 (-10.58 – 11.35) | -12.09 (-20.83 – -3.35) ** |

* p < 0.05; ** p < 0.01; *** p < 0.001

Interpretative key: This table presents the event study estimates of the effect of incident stroke on the rate of decline in cognitive function (assessed using TICS-m score). The reference period is the survey wave immediately prior to the wave in which stroke was self-reported by study participants. High definition fixed effects estimation method was used to fit all models, which accounts for all time-invariant confounders, including baseline age, race, sex and years of education. All models were adjusted for time-varying confounders, including marital status, CES-D score, and self-reported diagnosis of hypertension, diabetes, cancer, heart disease and psychiatric illness (estimates for the effects of these covariates are provided in supplementary table 2). A negative coefficient estimate indicates acceleration of cognitive decline relative to the rate in the reference period. Thus, in the overall sample, the rate of decline in cognitive function increased by 4.2 percentage points in the next wave after experiencing stroke relative to the rate of decline in the reference period.
