# Supplementary material for: Sex differences in post-stroke cognitive decline: A population-based longitudinal study of nationally representative data
Source: PLoS One. 2022 May 6;17(5):e0268249. doi: 10.1371/journal.pone.0268249 (PMC9075630; doi:10.1371/journal.pone.0268249)
Supplement: S3 Table — (DOCX) [file pone.0268249.s013.docx]

S3 Table: Event study coefficients for other time-varying covariates

| **Variable** | **Overall** | **Males** | **Females** |
| --- | --- | --- | --- |
| **Marital Status** |  |  |  |
| **Single** | Reference | Reference | Reference |
| **Married or partnered** | 0.63 (-4.12 – 5.37) | -2.43 (-8.89 – 4.03) | 2.23 (-4.24 – 8.71) |
| **Divorced or Separated** | 0.92 (-3.86 – 5.71) | -1.03 (-7.68 – 5.61) | 2.07 (-4.40 – 8.54) |
| **Widowed** | 0.09 (-4.71 – 4.89) | -5.08 (-11.75 – 1.58) | 2.56 (-3.93 – 9.06) |
| **Hypertension** | 0.47 (-0.12 – 1.06) | -0.12 (-1.01 – 0.78) | 0.87 (0.09 – 1.66) * |
| **Diabetes** | 0.26 (-0.50 – 1.01) | 0.16 (-1.00 – 1.32) | 0.29 (-0.70 – 1.27) |
| **Cancer** | -0.11 (-0.93 – 0.72) | 0.65 (-0.55 – 1.85) | -0.96 (-2.09 – 0.18) |
| **Heart Disease** | -0.76 (-1.42 – -0.09) * | -0.49 (-1.50 – 0.53) | -0.94 (-1.81 – -0.07) * |
| **Psychiatric Conditions** | -0.29 (-1.20 – 0.62) | -0.72 (-2.39 – 0.94) | -0.12 (-1.20 – 0.95) |
| **CESD Score** | -0.61 (-0.75 – -0.46) *** | -0.52 (-0.77 – -0.27) *** | -0.64 (-0.82 – -0.46) *** |

* p < 0.05; ** p < 0.01; *** p < 0.001
