# Supplementary material for: Sex differences in post-stroke cognitive decline: A population-based longitudinal study of nationally representative data
Source: PLoS One. 2022 May 6;17(5):e0268249. doi: 10.1371/journal.pone.0268249 (PMC9075630; doi:10.1371/journal.pone.0268249)
Supplement: S1 Appendix — (DOCX) [file pone.0268249.s014.docx]

**Details on data availability and ethics statement**

**Data Availability**

The data we used for this study was obtained from the Health and Retirement Study (HRS), which does not allow sharing and/or redistribution of its data products. However, the data is publicly available upon request and online registration at <https://hrsdata.isr.umich.edu/data-products/rand-hrs-longitudinal-file-2018>. The registration process is simple, fast, and only requires users to provide their name and contact information, as well as agree to the conditions of use for HRS public release data. The registration information can be found here: <https://hrsdata.isr.umich.edu/user/register?destination=node/38308>.

**Informed Consent**

As documented in the HRS IRB information document (which can be found here: https://hrs.isr.umich.edu/sites/default/files/biblio/HRS_IRB_Information-10-2017.pdf). HRS participants are provided with a written informed consent prior to each interview.
