# Supplementary material for: Sex differences in post-stroke cognitive decline: A population-based longitudinal study of nationally representative data
Source: PLoS One. 2022 May 6;17(5):e0268249. doi: 10.1371/journal.pone.0268249 (PMC9075630; doi:10.1371/journal.pone.0268249)
Supplement: S2 Appendix — (DOCX) [file pone.0268249.s015.docx]

**S3 Appendix - Extended methods**

The event study model is one of the extensions of the difference-in-differences (DiD) model. In the canonical DiD model, there are two groups, the treatment and control group, and two time periods: pretreatment and posttreatment periods. The treatment group only receives treatment in the post-treatment period and the control group does not receive treatment in either of the two periods. Researchers then estimate the average treatment effect on the treated by comparing the average difference in pretreatment and post-treatment outcomes between treated and control units or by using the two-way fixed effects regression model with fixed effect for group and time. The validity of DiD in producing causal estimates of the effects of a treatment or an intervention depends on the parallel trends assumption, which presumes that the average outcome for the treatment and control group would evolve in parallel in the absence of treatment.

The event study design is one of the extensions of the canonical DiD model that accounts for presence of multiple pre- and post-intervention periods, as well as variations in the timing of treatment or intervention. In this study, our main intervention (independent) variable is self-reported history of incident stroke and our outcome variable is the rate of decline in cognitive function, which we measured using the modified version of the Telephone Interview for Cognitive Status (TICS-m) score. Our event study model (below) estimates the effect of incident stroke on the rate of decline in cognitive function while accounting for time-varying confounders, such as hypertension, diabetes, cancer, heart disease, psychiatric illnesses and depression score, and time-invariant confounders, such as baseline age, sex, race/ethnicity and years of education:

*Cognitive_Decline_it_ = α + β8(Lag 8_plus)_it_ + β7(Lag 7)_it_ + ………… + β2(Lag 2)_it_ + γ0(Lead 0)_it_ + …….. + γ8(Lead 8)_it_ + 𝐗****_it_****𝚪 + µ****_i_*** *+ λ****_t_*** *+ ε****_it_***

Where Cognitive_Decline_it_ is the rate of decline in cognitive function (in percentage points) for individual *i* at HRS wave *t*. We measured the rate of decline in cognitive function as the natural log of TICS-m score in a given HRS wave minus the natural log of TICS-m score in the preceding HRS wave, multiplied by 100; i.e. ln (*TICS-m****_it_****) − ln(TICS-m****_i(t-1)_***) × 100. Of note, this log-differencing stabilizes our model with respect to age-related decline in cognitive function.

Leads and lags were considered relative to the reference period, which is the HRS wave immediately preceding the wave in which stroke was self-reported. Thus, the coefficient of lead *t*, for example, represents the effect of incident stroke on the rate of cognitive decline t waves (t x 2 years) after the wave in which incident stroke was self-reported (note that Lead 8_plus is an accumulation of the effect of incident stroke on rate of cognitive decline 8 or more waves 16 or more years] after stroke). Conversely, the coefficient of lag t, for example, captures the difference between the rate of cognitive decline in the reference period and the rate of decline t waves (periods) before incident stroke (note that Lag 8_plus is an accumulation of the effect of incident stroke on rate of cognitive decline 8 or more waves before stroke). Small and non-significant coefficients for all lags may imply that parallel trends assumption was not violated. Of note, we observed a few significant pre-stroke coefficients in our event study analyses; however, these significant pre-stroke coefficients indicated improvement in cognitive function before stroke (positive coefficients), and, thus, will most likely bias our analysis towards the null.

The coefficient µ**_i_** is the individual fixed-effect (dummy variables representing each individual), which controls for observed and unobserved time-invariant confounders such as baseline age, sex, race/ethnicity, and baseline level of education, among others. Furthermore, λ**_t_** are HRS wave dummies accounting for wave-by-wave trends in the rate of cognitive decline across individuals. Finally, 𝐗**_it_**𝚪 represents time-varying covariates, including marital status, self-reported diagnosis of hypertension, diabetes, cancer, heart disease and psychiatric illness, as well as CES-D score.

We fit our event study model using the *eventdd* package in Stata. Details of the *eventdd* package can be found here: <http://www.damianclarke.net/research/papers/panelEvent.pdf>
