# Supplementary material for: Sex differences in post-stroke cognitive decline: A population-based longitudinal study of nationally representative data
Source: PLoS One. 2022 May 6;17(5):e0268249. doi: 10.1371/journal.pone.0268249 (PMC9075630; doi:10.1371/journal.pone.0268249)
Supplement: S3 Appendix — (DOCX) [file pone.0268249.s016.docx]

**S4 Appendix: A stylized example of variable coding for event study lags and leads**

| **Participant** | **Wave** | **Stroke Wave** | **Time to event** | **Lag 8_plus** | **Lag 7** | **Lag 6** | **Lag 5** | **Lag 4** | **…** | **Lead 0** | **Lead 1** | **Lead 2** | **…** | **Lead 7** | **Lead 8_Plus** |
| --- | --- | --- | --- | --- | --- | --- | --- | --- | --- | --- | --- | --- | --- | --- | --- |
| A | 1 | 6 | -5 | 0 | 0 | 0 | 1 | 0 | … | 0 | 0 | 0 | … | 0 | 0 |
| A | 2 | 6 | -4 | 0 | 0 | 0 | 0 | 1 | … | 0 | 0 | 0 | … | 0 | 0 |
| A | 3 | 6 | -3 | 0 | 0 | 0 | 0 | 0 | … | 0 | 0 | 0 | … | 0 | 0 |
| A | 4 | 6 | -2 | 0 | 0 | 0 | 0 | 0 | … | 0 | 0 | 0 | … | 0 | 0 |
| A | 5 | 6 | -1 | 0 | 0 | 0 | 0 | 0 | … | 0 | 0 | 0 | … | 0 | 0 |
| A | 6 | 6 | 0 | 0 | 0 | 0 | 0 | 0 | … | 1 | 0 | 0 | … | 0 | 0 |
| A | 7 | 6 | 1 | 0 | 0 | 0 | 0 | 0 | … | 0 | 1 | 0 | … | 0 | 0 |
| A | 8 | 6 | 2 | 0 | 0 | 0 | 0 | 0 | … | 0 | 0 | 1 | … | 0 | 0 |
| A | 9 | 6 | 3 | 0 | 0 | 0 | 0 | 0 | … | 0 | 0 | 0 | … | 0 | 0 |
| A | 10 | 6 | 4 | 0 | 0 | 0 | 0 | 0 | … | 0 | 0 | 0 | … | 0 | 0 |
| A | 11 | 6 | 5 | 0 | 0 | 0 | 0 | 0 | … | 0 | 0 | 0 | … | 0 | 0 |
| B | 1 | 2 | -1 | 0 | 0 | 0 | 0 | 0 | … | 0 | 0 | 0 | … | 0 | 0 |
| B | 2 | 2 | 0 | 0 | 0 | 0 | 0 | 0 | … | 1 | 0 | 0 | … | 0 | 0 |
| B | 3 | 2 | 1 | 0 | 0 | 0 | 0 | 0 | … | 0 | 1 | 0 | … | 0 | 0 |
| B | 4 | 2 | 2 | 0 | 0 | 0 | 0 | 0 | … | 0 | 0 | 1 | … | 0 | 0 |
| B | 5 | 2 | 3 | 0 | 0 | 0 | 0 | 0 | … | 0 | 0 | 0 | … | 0 | 0 |
| B | 6 | 2 | 4 | 0 | 0 | 0 | 0 | 0 | … | 0 | 0 | 0 | … | 0 | 0 |
| B | 7 | 2 | 5 | 0 | 0 | 0 | 0 | 0 | … | 0 | 0 | 0 | … | 0 | 0 |
| B | 8 | 2 | 6 | 0 | 0 | 0 | 0 | 0 | … | 0 | 0 | 0 | … | 0 | 0 |
| B | 9 | 2 | 7 | 0 | 0 | 0 | 0 | 0 | … | 0 | 0 | 0 | … | 1 | 0 |
| B | 10 | 2 | 8 | 0 | 0 | 0 | 0 | 0 | … | 0 | 0 | 0 | … | 0 | 1 |
| B | 11 | 2 | 9 | 0 | 0 | 0 | 0 | 0 | … | 0 | 0 | 0 | … | 0 | 1 |
| C | 1 | 10 | -9 | 1 | 0 | 0 | 0 | 0 | … | 0 | 0 | 0 | … | 0 | 0 |
| C | 2 | 10 | -8 | 1 | 0 | 0 | 0 | 0 | … | 0 | 0 | 0 | … | 0 | 0 |
| C | 3 | 10 | -7 | 0 | 1 | 0 | 0 | 0 | … | 0 | 0 | 0 | … | 0 | 0 |
| C | 4 | 10 | -6 | 0 | 0 | 1 | 0 | 0 | … | 0 | 0 | 0 | … | 0 | 0 |
| C | 5 | 10 | -5 | 0 | 0 | 0 | 1 | 0 | … | 0 | 0 | 0 | … | 0 | 0 |
| C | 6 | 10 | -4 | 0 | 0 | 0 | 0 | 1 | … | 0 | 0 | 0 | … | 0 | 0 |
| C | 7 | 10 | -3 | 0 | 0 | 0 | 0 | 0 | … | 0 | 0 | 0 | … | 0 | 0 |
| C | 8 | 10 | -2 | 0 | 0 | 0 | 0 | 0 | … | 0 | 0 | 0 | … | 0 | 0 |
| C | 9 | 10 | -1 | 0 | 0 | 0 | 0 | 0 | … | 0 | 0 | 0 | … | 0 | 0 |
| C | 10 | 10 | 0 | 0 | 0 | 0 | 0 | 0 | … | 1 | 0 | 0 | … | 0 | 0 |
| C | 11 | 10 | 1 | 0 | 0 | 0 | 0 | 0 | … | 0 | 1 | 0 | … | 0 | 0 |
| D | 1 | . | . | 0 | 0 | 0 | 0 | 0 | … | 0 | 0 | 0 | … | 0 | 0 |
| D | 2 | . | . | 0 | 0 | 0 | 0 | 0 | … | 0 | 0 | 0 | … | 0 | 0 |
| D | 3 | . | . | 0 | 0 | 0 | 0 | 0 | … | 0 | 0 | 0 | … | 0 | 0 |
| D | 4 | . | . | 0 | 0 | 0 | 0 | 0 | … | 0 | 0 | 0 | … | 0 | 0 |
| D | 5 | . | . | 0 | 0 | 0 | 0 | 0 | … | 0 | 0 | 0 | … | 0 | 0 |
| D | 6 | . | . | 0 | 0 | 0 | 0 | 0 | … | 0 | 0 | 0 | … | 0 | 0 |
| D | 7 | . | . | 0 | 0 | 0 | 0 | 0 | … | 0 | 0 | 0 | … | 0 | 0 |
| D | 8 | . | . | 0 | 0 | 0 | 0 | 0 | … | 0 | 0 | 0 | … | 0 | 0 |
| D | 9 | . | . | 0 | 0 | 0 | 0 | 0 | … | 0 | 0 | 0 | … | 0 | 0 |
| D | 10 | . | . | 0 | 0 | 0 | 0 | 0 | … | 0 | 0 | 0 | … | 0 | 0 |
| D | 11 | . | . | 0 | 0 | 0 | 0 | 0 | … | 0 | 0 | 0 | … | 0 | 0 |
